# Effect of carbon on the co-presence of metallic tungsten as a nucleation agent and Eu^2+^ in glass: crystallization of CaO–Al_2_O_3_–SiO_2_ glass probed with Eu^2+^ luminescence

**DOI:** 10.1039/d2ra05766b

**Published:** 2022-11-03

**Authors:** Shingo Machida, Naoki Emori, Ken-ichi Katsumata, Kei Maeda, Atsuo Yasumori

**Affiliations:** Department of Material Science and Technology, Faculty of Advanced Engineering, Tokyo University of Science 6-3-1 Niijuku, Katsushika-ku Tokyo 125-8585 Japan shingo.machida@rs.tus.ac.jp

## Abstract

This study demonstrated simple redox control in glasses by improving the method used to added glass raw materials. Specifically, the effect of carbon on the co-presence of metallic tungsten (W) particles as nucleation agents and Eu^2+^ ions in CaO–Al_2_O_3_–SiO_2_ (CAS) glass was investigated *via* their crystallization to form CAS glass-ceramics (GCs). In this study, the glass specimens were prepared by mixing glass cullet containing metallic W particles and Eu^2+^ ions, respectively, with a glass batch containing carbon. Whereas the glass specimen was yellowish because of the presence of Eu^2+^ when carbon was not added during the remelting process, the glass specimen prepared with carbon was black because of the presence of metallic W particles. In addition, this specimen displayed the 470 nm emission band in its fluorescence spectrum recorded under 393 nm excitation, which was attributed to the presence of Eu^2+^. According to the fluorescence and transmission spectra, the glass specimen showed a darker coloration and more intense 470 nm emission band compared with the specimen prepared by the conventional melting method that included a remelting process. These results indicated that metallic W and Eu^2+^ were reduced with greater efficiency by the melting method that involved mixing the glass cullet and batch. In addition, the heat-treated glass specimen prepared by the aforementioned mixing method contained a greater amount of metastable CaAl_2_Si_2_O_8_ with increasing heat treatment time as revealed by X-ray diffraction analysis and scanning electron microscopy observation. The intensity of the 470 nm emission band decreased with increasing intensity of the band at 420 nm because of the incorporation of Eu^2+^ into the crystalline phase, and the increase in intensity of the 420 nm band was lineally proportional to the volume fraction of the crystallized glass specimens. The results therefore indicated that the co-presence of metallic W particles as nucleation agents and Eu^2+^ as a probe for tracking the crystallization process was achieved by the addition of carbon during the remelting process of mixed cullet containing W and Eu^2+^ through crystallization of the CAS glass. The results thus demonstrate the importance of improving the method used to added glass raw materials.

## Abstract 2

Effect of carbon on the co-presence of metallic tungsten (W) as nucleation agents and Eu^2+^ ions as a probe for tracking crystallization in CaO–Al_2_O_3_–SiO_2_ (CAS) glass was demonstrated by efficient reduction method of Eu^2+^ and metallic W.

## Graphical abstract

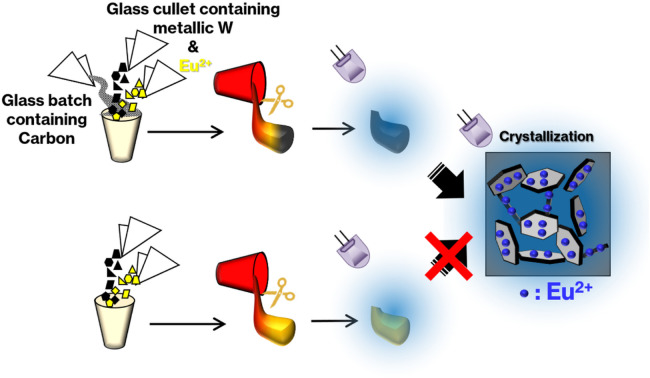

## Introduction

Composite materials that comprise two or more components have displayed chemical and physical properties that differ from those of the individual components. In addition, composites can compensate for the shortcoming of individual components and make them functional. One example is glass-ceramics (GCs)^1–9^ generated by precipitating crystalline phases in glassy phases. GCs were developed because glass bulk materials are brittle materials whose mechanical properties are improved by the precipitation of crystal particles.^1–5^ In the case where the crystal particles exhibit luminescence, the corresponding GCs are considered to be highly promising optical materials.^2–9^ These GCs can be obtained by crystallization of glass doped with rare-earth ions such as Eu^3+^, Tb^3+^, and Dy^3+^.^6–9^ Some of GCs exhibit a decrease in the luminescence with increasing crystal field.^9–11^ In contrast to these rare-earth ions, Eu^2+^ can exhibit an increase in luminescence with increasing crystal fraction,^12–16^ which might be advantageous for using spectroscopic imaging.^17,18^ In addition to rare-earth ions being used as dopants in GCs as optical materials, the exhibit luminescence properties that make them useful for probing crystallization processes.^10^ Therefore, Eu^2+^ luminescence can potentially be used to assess the crystal fraction in GCs, which is advantageous because the methods used to characterize the crystallization phases of GCs are basically X-ray diffraction (XRD) and microscopy. Thus, from the viewpoint of Eu^2+^ luminescence versatility, the oxidation of Eu^2+^ during the melting stage^10^ and/or during heat treatment of a glass should be appropriately suppressed depending on the glass compositions.

Herein, we devote special attention to simple redox control in glass by improving the addition method for glass raw materials. Specifically, this study investigates the effect of carbon on the co-presence of metallic tungsten (W) particles as nucleation agents^19–21^ and Eu^2+^ in glass. Metallic W and molybdenum (Mo) particles are critical nucleation agents for preparing CaO–Al_2_O_3_–SiO_2_ (CAS) GCs with precipitated metastable CaAl_2_Si_2_O_8_, a layered crystal with a hexagonal platy shape (CAS GC-H) forming a house-of-cards structure^10,19–26^ in glass similar to that of machinable mica GCs.^24^ In contrast to mica GCs, this CAS GC displays relatively high transparency despite the precipitation as micron-sized layered crystals;^19,25^ this transparency is advantageous in fundamental luminescence studies.^10^ Concerning the precipitation of metallic W and Mo particles in the CAS glass, tungsten oxide (WO_3_) and molybdenum oxide (MoO_3_) are reduced by carbon during glass melting.^10,19–25^ In our previous research, metallic Mo particles, whose oxidation free energy is similar to that for metallic W particles^27^ were absent in the CAS glass with a yellowish color caused by Eu^2+^ when the glass raw material containing MoO_3_, carbon, and europium oxide (Eu_2_O_3_) was melted, suggesting that metallic Mo was oxidized when Eu^3+^ was reduced.^10^ The efficient reduction of both Eu^3+^ and MoO_3_ is thus necessary. In previous related reports, meanwhile, Mo clusters in glass were reported to result in yellowish color^28^ that can overlap the Eu^2+^-induced coloration. Therefore, improving the method used to added W to glass is the best strategy in cases where Eu^2+^ and metallic W particles are co-present in CAS glass. Thus, in the present study, we mixed of glass cullet containing metallic W particles and Eu^2+^, and then the melted cullet with glass batch containing carbon, exploring the appropriate amounts of carbon through the crystallization of CAS glass. In addition, we compared the proposed method with the conventional melting method containing remelting process. Notably, in our previous studies,^19–21^ we found that the additives and their amount strongly affect the remelting processes.

## Experimental

In the present study, 50 g of CAS glass specimens was prepared by heating the raw materials at 1550 °C for 1 h under air in an alumina crucible.^10,16,19,20^ All the raw materials were reagent grade and were obtained from Wako Pure Chemical. Carbon was obtained from Kojundo Chemcal Laboratory.^20^ The nominal composition of the glass was 25CaO–20Al_2_O_3_–55SiO_2_ (wt%)^16^ where calcium carbonate (CaCO_3_) was used as a CaO source. The glass batches with this composition, along with 0.80 wt% WO_3_ and 4.0 wt% C, or 1.6 wt% Eu_2_O_3_ and 4.0 wt% C were melted to form glass cullet containing metallic W particle (Cullet-W) or Eu^2+^ ions (Cullet-Eu^2+^), respectively. The same weight of this glass cullet and the same compositional glass batch with 4.0, 0.40, or 0 wt% C were mixed and then melted to form three glass specimens with a composition of 25CaO–20Al_2_O_3_–55SiO_2_ (wt%) with 0.27wt% WO_3_ and 0.53wt% Eu_2_O_3_ (denoted herein as W-Eu-Glass-*x*C where *x* represents the carbon content (wt%)). For comparison, the conventional melting method was also used: a glass batch with a composition of 25CaO–20Al_2_O_3_–55SiO_2_ (wt%) with 0.27 wt% WO_3_, 0.53 wt% Eu_2_O_3_, and 4.0 wt% C was melted twice, where the remelting process consisted of adding a glass batch with same composition and weight; the resultant glass specimen is designated herein as W-Eu-Glass-normal. These glass specimens were crystallized by heat treatment at 1050 °C for 0, 2, or 6 h with heating and cooling rates of 100 °C min^−1^ according to the previous studies,^16,20^ to generate the glass specimens referred to herein as W-Eu-Glass-*x*C-*y*h where *y* represents the heat treatment time. Notably, a 0 h heat treatment indicates that the glass was subjected to the aforementioned elevated and reduced temperatures. Each glass specimen was cut and polished to remove the surface layer and to obtain glass specimens with a consistent size for the analyses described in the following paragraph. Note that the surface layer was drastically increased when the present type of glass specimen was heat-treated at 1100 °C. The effect of chemical composition on the luminescence property was investigated by performing, the glass specimen was prepared by the melting of the glass batch with the composition of 27CaO–13Al_2_O_3_–60SiO_2_ (wt%) with 0.53wt% Eu_2_O_3_ and 4.0 wt% C whose major composition was assumed and roughly estimated by the 30 wt% precipitation of metastable CaAl_2_Si_2_O_8_ in the CAS glass as described in our previous report.^10^ The glass specimen was denoted herein as Eu-Al-low-Glass. For the comparison of the luminescence properties of glass specimens after heat treatment with those of the crystalline product, the solid-state sample was prepared by mechanically grinding purified and expanded kaolinite (Al_2_Si_2_O_5_(OH)_4_) and CaCO_3_ and heating the mixtures under a N_2_ atmosphere containing 3 vol% H_2_ (*i.e.*, a reductive atmosphere) at 900 °C for 12 h.^29,30^ Notably, the solid-state reaction of kaolinite and CaCO_3_ forms anorthite (CaAl_2_Si_2_O_8_) and metastable CaAl_2_Si_2_O_8_ depending on the reaction temperature and atmosphere.^30^ The obtained product was thus denoted as Eu^2+^–CaAl_2_Si_2_O_8_ for convenience.

The crystalline phases of the glass specimens and the solid-state product were characterized by XRD analysis (XRD-610, Shimadzu). The microstructures of the glass specimens after heat treatment were characterized by scanning electron microscopy (SEM:TM-3000, Hitachi). The crystal volume fractions (vol%) in the heat-treated glass specimens were approximately using binarized SEM images. Fluorescence spectra of the glass specimens before and after crystallization, as well as the spectrum of solid-state product were obtained with excitation at 393 or 463 nm (FP-6500, JASCO). The thickness of the glass specimens for fluorescence analysis was 1 mm.

## Results and discussion

Notably, W cluster have previously been reported to result in bluish phosphate glass.^28^ However, such coloration was not observed in the colorless CAS glass prepared by the oxidative melting of glass cullet containing metallic W particles in our previous study.^19,21^ Therefore, the oxidation of metallic W particles in the CAS glass with the present composition likely dose not generate a glass specimen with W cluster coloration. Meanwhile, the colorless glass that never showed bulk crystallization was obtained when the raw material of CAS glass was melted without addition of carbon.^16^ In addition, it is revealed that metallic particles are acted as heterogeneous nucleation sites for crystallization of metastable CaAl_2_Si_2_O_8_ based on transmission electron microscopy.^17^ Furthermore, various coloration of glasses containing metallic W particles exhibited crystallization of metastable CaAl_2_Si_2_O_8_.^19–21^ Therefore, metallic W particles likely act as heterogeneous nucleation sites.

**Fig. 1:**
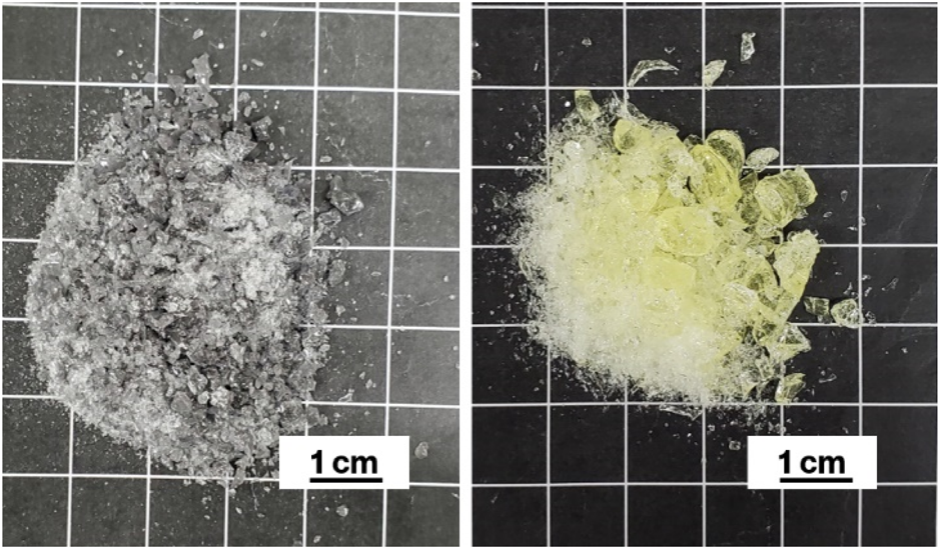
Photographs of Cullet-W (left) and Cullet-Eu^2+^ (right).

**Fig. 2:**
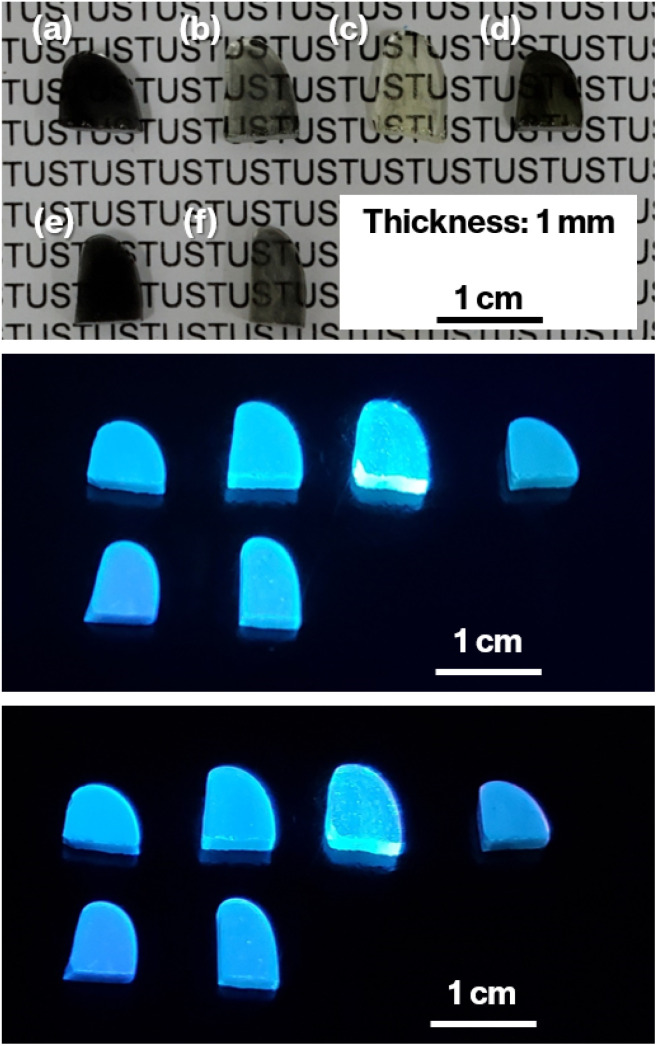
Photographs of (a) W-Eu-Glass-4.0C, (b) W-Eu-Glass-0.40C, (c) W-Eu-Glass-0C, (d) W-Eu-Glass-normal, (e) W-Eu-Glass-4.0C-2h, and (f) W-Eu-Glass-0.40C-2h under normal lighting (top), under 254 nm irradiation (middle), and under 365 nm irradiation (bottom).

**Fig. 3:**
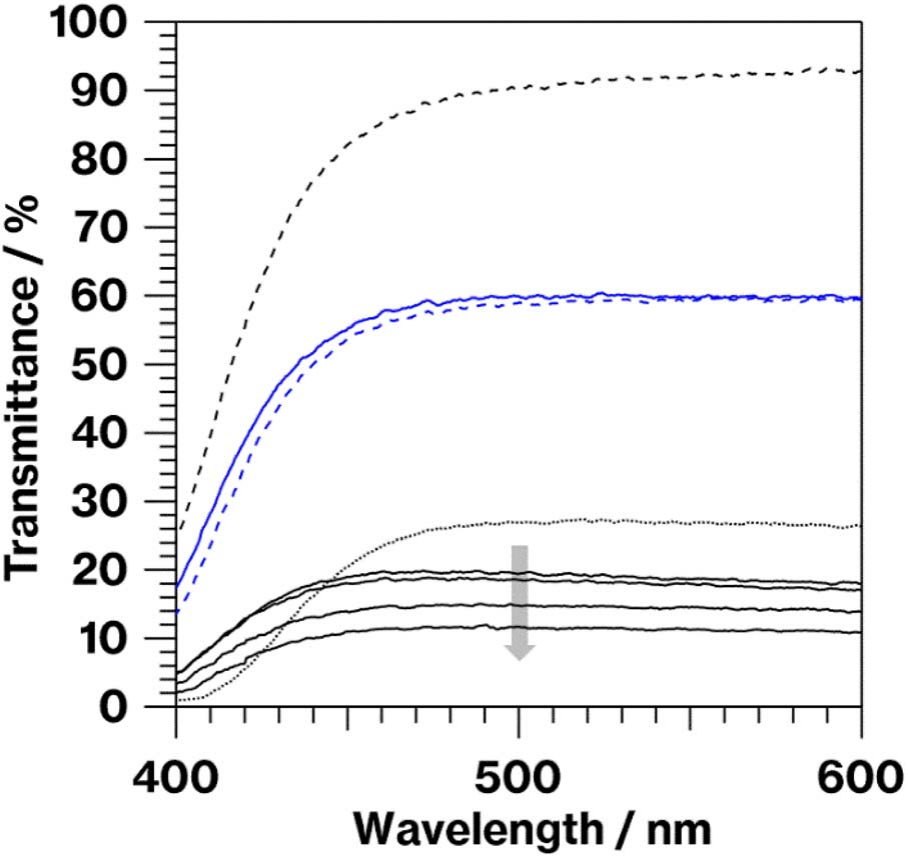
Transmission spectra of W-Eu-Glass-4.0C, W-Eu-Glass-4.0C-0h, W-Eu-Glass-4.0C-2h, W-Eu-Glass-4.0C-6 h (black solid lines in order of gray arrow), W-Eu-Glass-0.40C (blue solid line), W-Eu-Glass-0.40C-2h (blue dashed line), W-Eu-Glass-0C (black dashed line), and W-Eu-Glass-normal (black dotted line).

Cullet-W appeared black because of the presence of metallic W particles (Fig. 1, left), in good agreement with our previous studies.^19–21,24,27,31^ By contrast, Cullet-Eu^2+^ was yellowish (Fig. 1, right), consistent with the results of the previous studies in which Eu^2+^-doped glass specimens were reported to be yellow.^10,31^ These observations suggest that WO_3_ and Eu^3+^ were reduced by carbon burning at the glass melting stage. The black color of W-Eu-Glass-4.0C was similar to that of Cullet-W, whereas the black coloration decreased in the order of W-Eu-Glass-0.40C and -0C (Fig. 2, top). The color of W-Eu-Glass-0C was similar to that of Cullet-Eu^2+^. In our previous study, the oxidation of metallic particle upon the reduction of Eu^3+^ resulted in a yellowish glass specimen containing Eu^2+^.^10^ In addition, the CAS glass with such coloration was never crystallized by heat treatment.^10^ In our previous study, we estimated the amount presence of metallic W particles using transmission spectra, where a decrease in transmittance for black glass specimens indicated an increase in the amount of metallic W particles.^19,20^ The transmission spectra of W-Eu-Glass-0C shows a decrease in transmittance at 470 nm or shorter wavelengths because of yellow color of Eu^2+^ (Fig. 3).^11,32,33^ Compared with the transmittance at 470 nm or longer wavelength in the spectrum of W-Eu-Glass-0C, that in the spectra of W-Eu-Glass-0.40C and -4.0C decreases in the order W-Eu-Glass-0.40C and -4.0C (Fig. 3). This decrease in the transmittance for W-Eu-Glass-0.40C and -4.0C was similar to that observed in the spectrum of W-Eu-Glass-0h (Fig. 3). Therefore, in the present study, the metallic W particles are likely oxidized during the melting process of W-Eu-Glass-0C. Notably, the coloration of W-Eu-Glass-4.0C was darker than that of W-Eu-Glass-normal (Fig. 2, top) and the transmittance at wavelengths longer and shorter than 440 nm for W-Eu-Glass-normal is higher and lower than those in the corresponding regions of W-EU-Glass-4.0C, respectively (Fig. 3), suggesting that both metallic W particles and Eu^3+^ ions in W-Eu-Glass-4.0C exhibit a greater reduction efficiency than those in W-Eu-Glass-normal. However, the blue coloration due to Eu^2+^ fluorescence^32^ under 254 nm irradiation was not visually distinguished between the glass specimens before and after crystallization (Fig. 2, middle). In addition, such blue fluorescence was clearly observed under 365 nm irradiation (Fig. 2, bottom), which is commonly used to visually check the red fluorescence of Eu^3+^-doped glass. Notably, the transmittance for heat-treated W-Eu-Glass-4.0C specimens decreases with increasing heat-treatment time, whereas no difference in transmittance is observed between the spectra of W-Eu-Glass-0.40C before and after heat treatment (Fig. 3). We, therefore, characterized the luminescence properties of the glass specimens.

**Fig. 4:**
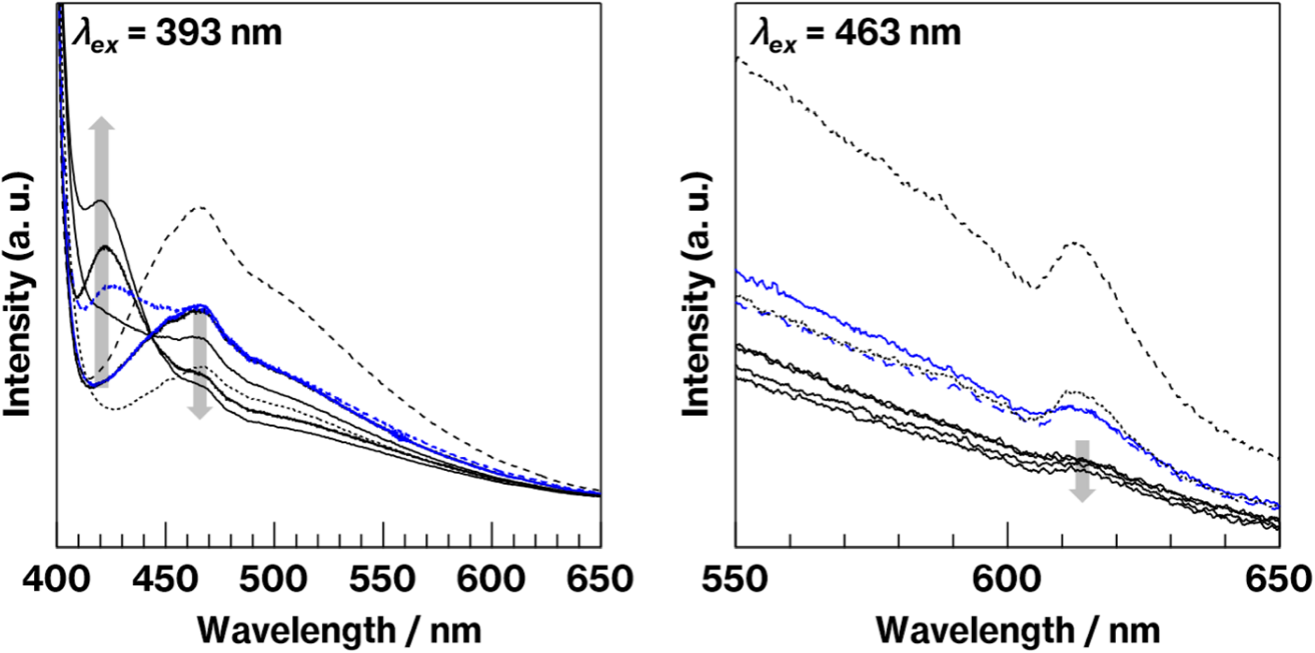
Fluorescence spectra of W-Eu-Glass-4.0C, W-Eu-Glass-4.0C-0h, W-Eu-Glass-4.0C-2h, W-Eu-Glass-4.0C-6 h (black solid lines in order of gray arrow), W-Eu-Glass-0.40C (blue solid line), W-Eu-Glass-0.40C-2h (blue dashed line), W-Eu-Glass-0C (black dashed line), and W-Eu-Glass-normal (black dotted line) under 393 nm (left) and 463 nm (right) excitation.

Fig. 4 shows fluorescence spectra of the glass specimens before and after crystallization. In the spectra acquired under 393 nm excitation, all the glass specimens exhibit broad emission bands at approximately 470 nm, which is attributed to Eu^2+^ fluorescence.^10–16,32,33^ The more intense emission at 470 nm is observed for W-Eu-Glass-0C, and W-Eu-Glass-4.0C and -0.40C show the same 470 nm emission intensity, which is stronger than that for W-Eu-Glass-normal. Compared with the spectra of glass specimens before the heat treatment, the spectra of heat-treated specimens exhibit weaker emission intensity at 470 nm and an increase in intensity at 420 nm with increasing the heat treatment time. In addition, the broad emission at 470 nm without the 420 nm emission was observed in the spectrum of Eu-Al-low Glass (data not shown). For 463 nm excitation, which is often used for exciting red Eu^3+^ luminescence,^34^ all the spectra exhibit weak Eu^3+^ fluorescence because of a ^5^D_0_ → ^7^F_2_ transition at 610 nm.^11^ In addition, the intensity of this fluorescence decreases in the order W-Eu-Glass-0C, -normal, -0.40C, and 4.0C. Among the samples, W-Eu-Glass-0.40C and -4.0C exhibit small changes in emission intensity after the heat treatment. Notably, the 610 nm emission was never observed in the fluorescence spectra recorded under 393 nm excitation, whereas in our previous study, broad emissions at both 470 and 610 nm were observed in the spectrum of yellowish CAS glass containing Eu^2+^ but not metallic Mo.^10^

**Fig. 5:**
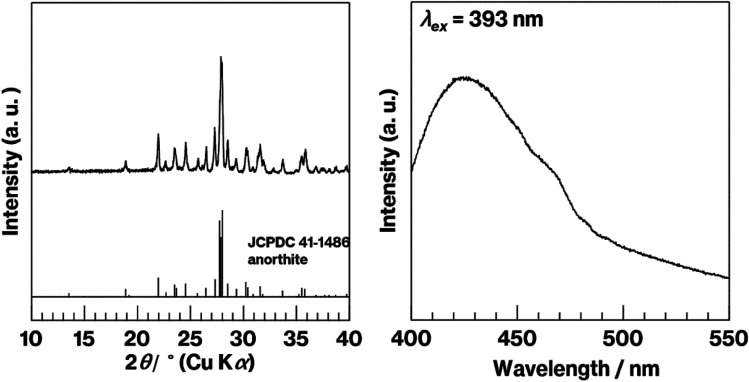
XRD pattern (left) and fluorescence spectrum (right) for Eu^2+^–CaAl_2_Si_2_O_8_.

In previous reports on Eu^2+^-doped GCs^12–16,33–35^ and phosphors whose mother materials were RAl_2_Si_2_O_8_ (where R represents an alkaline earth element),^36–40^ the 420 nm emission was clearly observed because of the incorporation of Eu^2+^ into the crystal structure. In the present study, reflections due to anorthite are observed in the XRD pattern of Eu^2+^–CaAl_2_Si_2_O_8_ and broad emission at 420 nm is observed in the fluorescence spectrum of Eu^2+^–CaAl_2_Si_2_O_8_ (Fig. 5, left and right). Notably, the solid-state reaction of kaolinite with CaCO_3_ forms metastable CaAl_2_Si_2_O_8_ at 900 °C under air but unfortunately generates anorthite (CaAl_2_Si_2_O_8_) under a reductive atmosphere despite the 900 °C reaction temperature.^30^ The preparation of Eu^2+^-doped metastable CaAl_2_Si_2_O_8_ could thus be the topic of a future study.

**Fig. 6:**
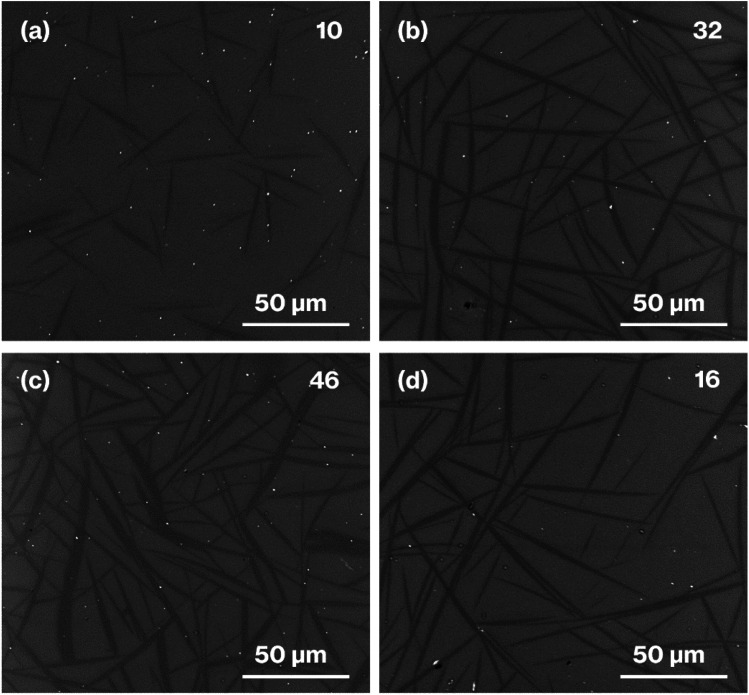
SEM images of (a) W-Eu-Glass-4.0C-0h, (b) W-Eu-Glass-4.0C-2h, (c) W-Eu-Glass-4.0C-6h, and (d) W-Eu-Glass-0.40C-2h. The volume fraction (vol%) is denoted in the upper right of each image.

Fig. 6 shows SEM images of the heat-treated glass specimens. The SEM images of the house-of-cards structures comprising hexagonal platy particles of metastable CaAl_2_Si_2_O_8_ show the black regions with a needle-like appearance that, according to our previous reports,^24–26^ correspond to an arbitrary cross-section of a house-of-cards structure representing the CAS GC-H microstructure. In the present study, needle-like particles are considered to be crystal particles for simplicity. Needle-like particles are observed in the SEM images of all the samples prepared in the present study. The SEM images of the heat-treated glass specimens show that the needle length and width, as well as volume fractions, increase with increasing heat treatment time. The limitation of the present study lies in the estimation of the aspect ratio^41,42^ of the needle-like particles, because an arbitrary cross-section of the house-of-cards structures includes the diagonal cross-sections of platy particles.

**Fig. 7:**
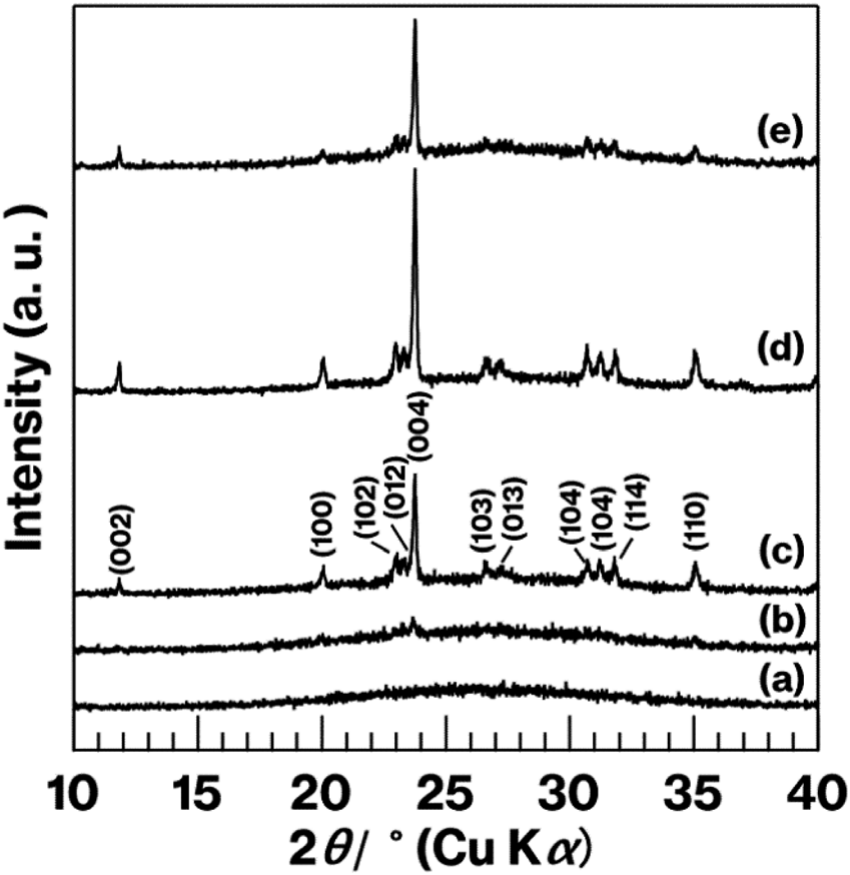
XRD patterns for (a) W-Eu-Glass-4.0C, (b) W-Eu-Glass-4.0C-0h, (c) W-Eu-Glass-4.0C-2h, (d) W-Eu-Glass-4.0C-6h, and (e) W-Eu-Glass-0.40C-2h.

Fig. 7 shows XRD patterns for the glass specimens. Unlike the pattern for W-Eu-Glass-4.0C, which shows a halo profile, the patterns for the heat-treated glass specimens show reflections attributed to metastable CaAl_2_Si_2_O_8_.^43^ The diffraction intensity increases with increasing heat treatment time in the patterns for the heat-treated W-Eu-Glass-4.0C. In addition, reflections due to calcium tungstate^44^ were not observed in any of the profiles, in agreement with our prior research.^19^ Notably, the (004) reflection is attributed to the stacking direction of aluminosilicate layers of metastable-CaAl_2_Si_2_O_8_.^43^ The intensity of the (004) reflection relative to that of other reflections is greater in the patterns for W-Eu-Glass-4.0C-6h and -0.40C-2h than in the pattern for W-Eu-Glass-4.0C-2h. In addition, as a result of greater volume fraction of W-Eu-Glass-4.0C-2h than W-Eu-Glass-0.40C-2h (Fig. 6b and d), the intensity of the (004) reflection looks like equivalent for these two glass specimens. Notably, XRD profiles vary depending on the crystallinity, stacking order, and lateral sizes of stacked inorganic layers composed of layered crystals;^45–47^ the intensity of reflections associated with the stacking direction relative to that associated with lateral atom arrangements is particularly variable.^29^

The results mentioned above, SEM images and XRD patterns (Fig. 6 and 7) indicate the precipitation of hexagonal platy particles of metastable CaAl_2_Si_2_O_8_ in the glass specimens after heat treatment. The coloration of the glass specimens under normal lighting and 254 nm irradiation (Fig. 2, top and middle), as well as their transmission and fluorescence spectra (Fig. 3 and 4), reveal the presence of Eu^2+^ in the glass specimens in with minute Eu^3+^ contents. The presence of the emission band at ∼420 nm in the fluorescence spectra of glass specimens after heat treatment, the position of which well matches that observed in the fluorescence spectrum of Eu^2+^–CaAl_2_Si_2_O_8,_ strongly implies that Eu^2+^ was incorporated into the crystalline phases in the glass specimens after the heat treatment. In addition, the appearance of 420 nm emission did not respond to the change of glass composition by crystallization based on the absence of 420 nm emission in the fluorescence spectra of W-Eu-Glass-0C and Eu-Al-low-Glass, indicating the responsibility of the 420 nm emission to the glass crystallization with concurrent Eu^2+^ accommodation to crystals. These results therefore indicate the formation of Eu^2+^-doped CAS GC-H in the heat-treated W-Eu-Glass-4.0C and -0.40C specimens. Given the absence of dark coloration in the yellowish W-Eu-Glass-0C (Fig. 2) whose color is the same as that of Eu^2+^-doped glass specimens,^10,32,33^ the carbon added during the remelting process suppressed the oxidation of both metallic W particles and Eu^2+^ ions in the mixed Cullet-W and Cullet-Eu^2+^ (Fig. 1), respectively. Thus, the co-presence of metallic particles as nucleation agents and Eu^2+^ ions in the CAS glass, which was not achieved in our prior research, was accomplished in the present study by the effect of carbon and by improving the addition method for glass raw materials.^10^ This interpretation fits well with the lines in Ellingham diagrams decreasing in the order W/WO_3_, EuO/Eu_3_O_4_, Eu_3_O_4_/Eu_2_O as reported in previous studies.^48,49^ Although the co-presence of metallic W and Eu^2+^ is evident in the glass specimen prepared by the conventional melting method, the reduction efficiency of this method is lower than that of the improved glass raw material addition method used in the present study, as evaluated on the bases of the coloration and the fluorescence spectra of glass specimens of W-Eu-Glass-4.0C, -0.40C, and -normal (Fig. 2 and 4). This greater reduction efficiency is attributed to the carbon combustion reaction being used to reduce WO_3_ and Eu_2_O_3_, which minimizes the oxidation of Eu^2+^ and metallic W. The amount of carbon used as a glass raw material appears to be sufficient for reducing WO_3_ and Eu_2_O_3_, although the increase in volume of the glass batch due to the carbon bulk resulted in the mixture hardly fitting in the crucible. In addition, to the best of our knowledge, EuO is not commercially available and commercial Eu^2+^ compounds contain halogens and sulfur whose effects on both the Eu^2+^ luminescence and nucleation processes should be considered; this is a topic of another detailed study. Meanwhile, although the transparency and coloration differ between W-Eu-Glass-4.0C, and -0.40C, the intensity of the 470 nm emission due to Eu^2+^ is the same for these two glass specimens that exhibit minute differences in the intensity of their Eu^3+^ emission. Moreover, the XRD patterns and SEM images show that the amounts of crystalline phases differ between W-Eu-Glass-4.0C-2h and -0.40C-2h (Fig. 6 and 7). Therefore, suppressing of Eu^2+^ oxidation by controlling the amount of nucleation agent achieved by varying the amount of carbon added during the remelting process. Notably, the amounts of crystalline phases generally depend on the number of nucleation agents.^19–21,50^ In the case of a low carbon content (*e.g.* 0.40 wt%), metallic W particles would also help suppress Eu^2+^ oxidation to reduce the number of nucleation agents. Because glass specimens with a larger amount of nucleation agents are likely advantageous for studying the crystallization process,^10^ we evaluated the Eu^2+^ probing ability on the basis of the crystallization of CAS GC-H using W-Eu-Glass-4.0C specimens.

**Fig. 8:**
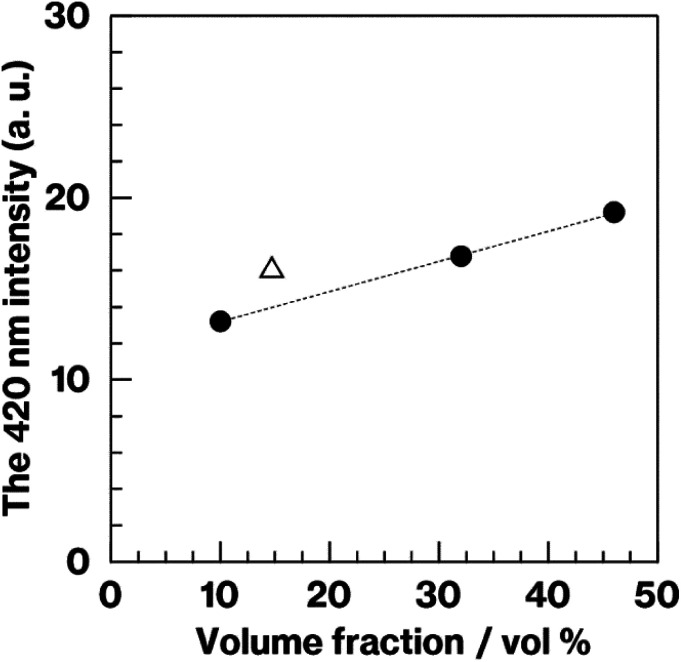
Intensity at 420 nm as a function of volume fraction (vol%) of crystalline phase, as obtained from the fluorescence spectra and SEM images of the heat-treated W-Eu-Glass-4.0C product (filled circles) and W-Eu-Glass-0.40C-2h (open triangle).

Fig. 8 shows the relationship between the 420 nm emission intensity and the volume fraction estimated from fluorescence spectra and SEM images of W-Eu-Glass-4.0C and -0.40C after crystallization. For W-Eu-Glass-4.0C, the emission intensity increases in proportion to the volume fraction of the crystalline phase, indicating that Eu^2+^ is successfully probed *via* the CAS glass crystallization. Given that the decrease in transparency upon crystallization of W-Eu-Glass-4.0C (Fig. 3) is likely due to light scattering by precipitating crystals, the Eu^2+^ luminescence in the W-Eu-Glass-4.0C specimens is unlikely affected by the presence of crystalline phases. The data point for W-Eu-Glass-0.40C-2h does not lie on the dotted line in Fig. 8. Based on the difference in coloration between the W-Eu-Glass-0.40C and -0.40C specimens (Fig. 2 and 3), the intensity of Eu^2+^ luminescence is likely affected by the dark coloration, which depends on the amount of metallic W particles. Unfortunately, however, a change in redox state of the metallic W particles during the heat treatment for crystallization is still unclear even in CAS GC-H.^19–25^ Therefore, a further study will be required to clarify the relationships among dark coloration, Eu^2+^ emission intensity, and crystallization, Ti^3+^, which can function as a reducer during glass heat treatment,^51^ is a feasible additive given that the lines for Ti_4_O_7_/TiO_2_ and Ti_3_O_5_/TiO_2_ lie below those for W/WO_3_ in the Ellingham diagrams.^52^ Meanwhile, in a previous study, a GC with precipitated CaAl_2_Si_2_O_8_, which was prepared *via* phase separation, displayed a slight shift of its 470 nm broad emission band upon crystallization. Although the 420 nm intensity increased after crystallization of BaO–Al_2_O_3_–SiO_2_–MgF_2_ glass, in which the main crystalline phase of was BaAl_2_Si_2_O_8_, the relationship between the increase intensity and crystallization is not linear according to previous reported results.^12^ In addition, by-phases are precipitated along with the main phase.^12^ These tendencies have been documented in the previous studies.^14,15^ Therefore, the present study clearly shows the linear relationships between the fluorescence intensity and crystallization amount because of the features of CAS GC-H, which has a single crystalline phase and relatively high transparency compared with GCs prepared *via* fluorine separation.^11,12,26^

In our previous study, we found that, during the remelting process, the additives and their amount strongly affected the amount of nucleation agents formed, thereby enabling the size of the hexagonal platy particles forming house-of-cards structures to be controlled in the 3–110 μm range.^19–21^ Among additives, carbon was added solely during the remelting process, resulting in the generation and an increase in the number of large platy particles due to the growth of metallic W particles.^19^ This interpretation explains the difference between W-Eu-Glass-4.0C-2h and W-Eu-Glass-0.40C-2h; the volume fraction and darkness of the former glass specimen are greater than those of the latter (Fig. 2, 3, and 6), suggesting the presence of larger and more metallic W particles in W-Eu-Glass-4.0C-2h than W-Eu-Glass-0.40C-2h. Thus, microstructural control and increased transparency of CAS GC-H is achieved under the presence of Eu^2+^. Among them, an increase in transparency of Eu^2+^-doped CAS GC-H could be useful for optimizing the luminescence property. Notably, we recently reported the effect of the particle size of carbon as a glass raw material on to both the reduction efficiency of MoO_3_ and microstructural control in CAS GC-H.^20^ The size of additives and the addition method for the glass batch and cullet were found to be key parameters for controlling both the redox states and microstructure in glass and GCs. These insights will be used for in further studies of rare-earth doped CAS and other types of glass and GC.

## Conclusions

We have demonstrated the effect of carbon on the co-presence of metallic W particles and Eu^2+^ ions in CAS glass by observing the crystallization of the CAS glass to form CAS GC-H, where the method of mixing cullet containing metallic W and Eu^2+^ with glass batch containing carbon in the remelting process efficiently suppressed the oxidation of metallic W particles and Eu^2+^, in contrast to the conventional melting method that includes a remelting process. In addition, the Eu^2+^-doped CAS GC-H enabled crystallization tracking using the Eu^2+^ luminescence as a probe, where the luminescence intensity increased in proportion of the crystal volume fraction. Furthermore, the microstructure of the materials investigated in present study was found to be closely related to that of CAS GC-H, which exhibits larger hexagonal platy particles that lead to improved microhardness.^19^ These results therefore demonstrated the potential to combine studies of mechanical properties^19–26^ and spectroscopy^17,18^ to develop Eu^2+^ luminescence versatility.

## Author contributions

Shingo Machida: conceptualization, writing—original draft, data curation, investigation, supervision. Naoki Emori: investigation. Kei Maeda: writing—review and editing. Ken-ichi Katsumata: project administration. Atsuo Yasumori: project administration.

## Conflicts of interest

There are no conflicts of interest.

## Supplementary Material

## References

[cit1] DeCeanne A. V., Rodrigues L. R., Wilkinson C. J., Mauro J. C., Zanotto E. D. (2022). J. Non-Cryst. Solids.

[cit2] Deubener J., Allix M., Davis M. J., Duran A., Höche T., Honma T., Komatsu T., Krüger S., Mitra I., Müller S., Nakane S., Pascual M. J., Schmelzeri J. W. P., Zanotto E. D., Zhou S. (2018). J. Non-Cryst. Solids.

[cit3] Höland W. and Beall G. H., Glass-Ceramic Technology, ed. W. Höland and G. H. Beall, Wiley-America Ceramic Society, Hoboken, New Jersey, 3rd edn, 2019, pp 67–357

[cit4] Allix M. and Cormier L., Crystallization and Glas-Ceramics, in Springer Handbook of Glass, ed. J. D. Musgraves, J. Hu and L. Calvez, Springer Nature, Switzerland, 1st edn, 2019, pp 113–168

[cit5] Homma T., Maeda K., Nakane S., Shinozaki K. (2022). J. Ceram. Soc. Jpn..

[cit6] Cruz M. E., Sedano M., Castro Y., Pascual M. J., Fernández J., Balda R., Durán A. (2022). Opt. Mater. Express.

[cit7] Kajihara K., Kanamori K., Shimojima A. (2022). J. Ceram. Soc. Jpn..

[cit8] Komatsu T., Honnma T. (2013). Int. J. Appl. Glass Sci..

[cit9] Cruz M. E., Castro Y., Durán A. (2022). J. Sol-Gel Sci. Technol..

[cit10] Machida S., Yamaguchi T., Emori N., Katsumata K., Maeda K., Yasumori A. (2022). Inorg. Chem..

[cit11] Taruata S., Matsuki M., Nishikiori H., Yamakami T., Yamaguchi T., Kunio K. (2010). Ceram. Int..

[cit12] Evstropiev S. K., Shashkin A. V., Knyazyan N. B., Manukyan G. G., Bagramyan V. V., Timchuk V., Stolyarova V. L. (2022). J. Non-Cryst. Solids.

[cit13] Rahimi M., Zahedifar M., Azimirad R., Faeghinia A. (2020). J. Lumin..

[cit14] Bouchouicha H., Panczer G., de Ligny D., Guyot Y., Baesso M. L., Andrade L. H. C., Lima S. M., Ternane R. (2016). J. Lumin..

[cit15] Herrmann A., Simon A., Rüseel C. (2012). J. Lumin..

[cit16] Biswas K., Sontakke A. D., Sen R., Annapurna K. (2012). J. Fluoresc..

[cit17] Zhang R., Ying Y., Rao X., Li J. (2012). J. Sci. Food Agric..

[cit18] Trovatello G., Genco A., Cruciano C., Ardini B., Li Q., Zhu X., Valentini G., Cerullo G., Manzoni C. (2022). Opt. Mater. X.

[cit19] Machida S., Murayama M., Maeda K., Katsumata K., Yasumori A. (2022). J. Ceram. Soc. Jpn..

[cit20] Machida S., Maeda K., Katsumata K., Yasumori A. (2022). Int. J. Appl. Glass Sci..

[cit21] Machida S., Maeda K., Katsumata K., Yasumori A. (2022). ACS Omega.

[cit22] Maeda K., Yasumori A. (2016). Mater. Lett..

[cit23] Maeda K., Yasumori A. (2017). Mater. Lett..

[cit24] Maeda K., Iwasaki K., Urata S., Akatsuka K., Yasumori A. (2019). J. Am. Ceram. Soc..

[cit25] Maeda K., Akatsuka K., Okuma G., Yasumori A. (2021). Crystals.

[cit26] Okuma G., Maeda K., Yoshida S., Takeuchi A., Wakai F. (2022). Sci. Rep..

[cit27] Maeda K., Yasumori A. (2015). J. Non-Cryst. Solids.

[cit28] Poirier G., Ottoboni F. S., Cassanjes F. C., Remonte Á., Messaddeq Y., Ribeiro S. J. L. (2008). J. Phys. Chem. B.

[cit29] Machida S., Katsumata K., Yasumori A. (2021). RSC Adv..

[cit30] Machida S., Katsumata K., Yasumori A. (2022). RSC Adv..

[cit31] Maeda K., Yasumori A. (2016). J. Non-Cryst. Solids.

[cit32] Watanabe S., Osawa Y., Machida S., Katsumata K., Yasumori A., Takahashi K., Deguchi K., Ohki S., Segawa H. (2021). J. Non-Cryst. Solids.

[cit33] Luo Q., Fan X., Qiao X., Yang H., Wang M., Zhang X. (2009). J. Am. Ceram. Soc..

[cit34] Żur L. (2013). J. Mol. Struct..

[cit35] Ohgaki T., Higashida A., Soga K., Yasumori A. (2007). J. Electrochem. Soc..

[cit36] Im W. B., Kim Y.-I., Jeon D. Y. (2006). Chem. Mater..

[cit37] Zhang L., Yamada H., Imai Y., Xu C.-N. (2008). J. Electrochem. Soc..

[cit38] Lu F. C., Bai L.-J., Yang B.-Z., Yang Z.-P. (2013). ECS J. Solid State Sci. Technol..

[cit39] Ma M., Zhu D., Zhao C., Han T., Cao S., Tu M. (2012). Opt. Commun..

[cit40] Sun L., Wnag Q., Zhang X., Yang Z., Cheng J., Sidike A., He J. (2020). J. Lumin..

[cit41] Machida S., Hashimoto R., Yoshida T., Ogawa M. (2014). J. Colloid Interface Sci..

[cit42] Ogawa M., Hiramine M. (2014). Cryst. Growth Des..

[cit43] Akatsuka K., Yasumori A., Maeda K. (2019). Mater. Lett..

[cit44] Otsuka T., Brehl M., Cicconi R. M., de Ligny D., Hayakawa T. (2021). Materials.

[cit45] Miyamoto N., Nakato T. (2012). Isr. J. Chem..

[cit46] Ogawa M., Saito K., Sohmiya S. (2014). Dalton Trans..

[cit47] Detellier C. (2018). Chem. Rec..

[cit48] Tominov R. V., Vakulov Z. E., Polupanov N., Saenko A., Avilov V. I., Ageev O. A., Smirnov V. A. (2022). Nanomaterials.

[cit49] Jacob K. T., Rajput A. (2016). J. Chem. Eng. Data.

[cit50] Stookey S. D. (1959). J. Int. Eng. Chem..

[cit51] Maeda K. and Yasumori A., JAP Patent 6769476, Oct. 14, 2020

[cit52] Yang J. J., Strachan J. P., Miao F., Zhang M.-X., Pickett M. D., Yi W., Ohlberg D. A. A., Medeiros-Ribeiro G., Williams R. S. (2011). Appl. Phys. A.

